# The implementation of a culturally tailored parenting support programme for Somali immigrant parents living in Sweden—A process evaluation

**DOI:** 10.1371/journal.pone.0274430

**Published:** 2022-09-14

**Authors:** Fatumo Osman, Ulla-Karin Schön, Marie Klingberg-Allvin, Renée Flacking, Malin Tistad

**Affiliations:** 1 Department of Health and Welfare, Dalarna University, Falun, Sweden; 2 Department of Social Work, Stockholm University, Stockholm, Sweden; Universität Wien: Universitat Wien, AUSTRIA

## Abstract

**Background:**

Parental support programmes aim to strengthen family functioning and the parent–child relationship and to promote the mental health of children and parents. However, there is a lack of knowledge on how parenting support programmes can be implemented for newly arrived immigrant parents. This process evaluation describes the implementation of a successful parenting programme for immigrant parents from Somalia and identifies key components of the implementation process with a focus on Reach, Adaptation, and Fidelity of Ladnaan intervention.

**Method:**

This process evaluation considered context, implementation and mechanism of impact, in accordance with the Medical Research Council’s guidance. Data were collected through focus group discussions, a questionnaire, attendance lists, field and reflection notes and observations of the sessions. The data were then analysed using content analysis and descriptive statistics.

**Results:**

Of the 60 parents invited to the parenting programme, 58 participated in the sessions. The study showed that involving key individuals in the early stage of the parenting programme’s implementation facilitated reaching Somali-born parents. To retain the programme participants, parents were offered free transportation. The programme was implemented and delivered as intended. A majority of the parents were satisfied with the programme and reported increased knowledge about children’s rights and the support they could seek from social services.

**Conclusions:**

This study illustrates how a parenting support programme can be implemented for Somali-born parents and provides guidance on how to attract immigrant parents to and engage them in participating in parenting support programmes.

## Background

Increased global displacement over the last few decades led to approximately one million refugees resettling in European countries by 2015. Most of the refugees came from Syria, Afghanistan, Eritrea and Somalia, fleeing war, conflict and persecution. This displacement is also called forced migration [1]. Sweden has one of the highest numbers of asylum seekers per capita in Europe, and the Somali population is one of the largest groups of migrants from Africa in Sweden [2]. Forced migration has a strong effect on the health of the individual [3], a family’s transition to the host country (i.e. acculturation) [4, 5] and parent–child relationships [6–8]. Several studies have addressed the challenges faced by immigrant parents due to forced migration and acculturation in their new home country [9, 10]. Acculturation is defined as the cultural transition or adoption that occurs when an individual comes to a new context or country [11]; this does not mean that the individual detaches from their own culture but adapts consciously and unconsciously to the host country’s culture. On a family level, challenges to acculturation include the lack of extended family, a change in roles and power conflicts between parents and children due to their different levels of acculturation [8, 12]. On a societal level, challenges to acculturation include a lack of understanding in terms of the parenting system in the new home country, fear that authorities will remove children from their families and a sense of being discriminated against by social services [7, 13, 14]. The immigrant parents’ fear of social services and sense of discrimination may hinder them from seeking support for their parenting [7, 13–15]. Forced migration and the acculturation process are challenges that may impact family dynamics, parenting and the parent–child relationship [9–12].

An earlier study showed that Somali immigrant parents believed that resettling in another country improved their relationship with their children and that they modified their parenting to fit their new context [15]. Immigrant parents have stressed the need for culturally tailored parenting support programmes to help them adapt their parenting role and skills in their new home country and to strengthen parent–child relationships [11, 15, 16]. Studies and reports have highlighted culturally tailored parenting support programmes as a means of reducing existing inequalities in health [17–19]. Parenting programmes tailored for Somali immigrant parents have been shown to strengthen the functioning of families and parent–child relationships and to promote the mental health of children and parents [20, 21]. Despite these benefits, studies have revealed numerous challenges when it comes to engaging, recruiting and retaining immigrant parents in parenting programmes [22–24]. A lack of cultural sensitivity in such programmes contributes to low participation and high dropout rates among immigrant parents [23, 25]. Most types of parenting programmes also lack cultural sensitivity and are delivered in the language of the host country [23]. From a human rights’ perspective, it is crucial for a parenting programme to be delivered in the participants’ native language and by people with a similar background and culture to the participants [6, 8, 15, 16].

### Ladnaan—A culturally tailored parenting intervention

In previous studies [20, 21], we developed a culturally tailored parenting programme (the Ladnaan intervention) and evaluated its effectiveness for Somali-born parents in Sweden in a randomised controlled trial. Ladnaan is the Somali word for ‘a sense of health and wellbeing’. In the present study (RCT), cultural tailoring refers to the social relevance and cultural sensitivity strategies employed in the parenting support programme to meet the specific needs of the target population. To ensure cultural tailoring, a qualitative study on how a parenting support programme could meet Somali immigrant parents’ needs and how such support could be implemented was conducted prior to the development of the intervention [15]. The findings of the qualitative study guided the design of the Ladnaan intervention. The intervention was based on the following two components: the Connect parenting programme [26] and societal information. The Connect parenting programme was chosen to meet parents’ need to improve their relationship with their children. The evidence-based programme is derived from attachment theory and aims to enhance the parent’s reflection on their child’s behaviour and needs [26]. The societal information was based on a qualitative study conducted prior to the development of the intervention [15] and was intended to specifically address parents’ need for knowledge about being a parent in Sweden (see further detail on the Ladnaan intervention in the method section).

Our trial findings showed that the Ladnaan intervention decreased children’s behavioural problems [20] and improved parents’ mental health and sense of parental competence two months after the programme [21]. These positive changes were maintained three years post-intervention [27]. Somali-born parents reported that the culturally tailored parenting programme strengthened their parenting and parent–child relationships [28]. Because of the proven benefits of the programme, it is crucial to explore its implementation strategies and how the target group was reached. These details can guide the future implementation of similar programmes and provide information to policymakers about the programme’s potential in other settings.

### Evaluation of implementation strategies

Implementing a culturally tailored parenting programme through social services may entail several challenges. As stated earlier, immigrant parents reported fear of social services, which prevented them from seeking the services’ support [7, 13–15]. In the field of implementation research, there is no gold standard for how to support the implementation of an evidence-based programme. However, clearly the implementation’s outcome will be affected by the characteristics of the intervention, contextual factors, the implementation process, the mechanism of impact and individuals’ self-efficacy in using the intervention [29]. Using process evaluation may increase understanding of what factors contributed to the success or failure of implementing a complex intervention [30]. Process evaluation does not replace but complements randomised trials. Implementation—the process of putting a programme into use [31]—involves dimensions such as context, reach, fidelity and adaptation [32]. Context is defined by the external factors that might influence the intervention’s implementation and outcomes. Context factors might involve organisational resources and how the population receives the intervention [30]. Reach is defined as the ability of a programme to engage its target audience and is crucial for the programme’s potential to impact public health [30, 32]. Programme fidelity is generally defined as the degree to which a programme is delivered as intended [33]. Knowledge of programme fidelity is crucial if conclusions are to be drawn about the programme’s outcomes. Although the importance of programme fidelity is widely accepted, relatively little attention has been given to clarifying the strategies used to implement a programme [34, 35]. Adaptation is defined as modifying or tailoring the intervention to the needs and characteristics of the target group. While there is often tension between fidelity and adaptation, adapting the intervention to the target groups can be crucial to the reach and relevance of the programme [32].

There is a need for guidance to support future implementation efforts of parenting programmes. Hence, it is urgent to properly explore and describe core components of successful implementation process. Therefore, this process evaluation aims to explore aspects of the implementation process of parenting support programme. More specifically, this study addresses the following research questions:

1. What were the contextual barriers to and facilitators for reaching and retaining the target group?
2. What were the contextual barriers to the group leaders’ participation?
3. To what extent did parents in the target group participate in the intervention?
4. To what extent was the Ladnaan intervention delivered as intended, and how did group leaders perceive the delivery?
5. What adaptions of the implementation strategy were made, and how was the strategy perceived by the group leaders and internal facilitators?
6. Were group leaders satisfied with the delivery of the intervention?
7. Were the participating parents satisfied with the intervention?

## Method

### Design

This process evaluation of the Ladnaan intervention, a culturally tailored parenting programme, was conducted in parallel with a RCT. Findings from the RCT has been reported previously [20, 21] and the focus in this paper is the implementation process. The process evaluation was guided by the framework developed for the Medical Research Council Guidance by Moore et al. [30, 32]. Three components—context, implementation and mechanism of impact—were used to describe the context for the implementation, how the implementation strategy was carried out and potential mechanisms for achieving implementation. In Table 1, we describe how each component of the process evaluation and the data sources to respond to the research questions. Using both qualitative and quantitative approaches, we bring together different factors linked to the implementation of the intervention [30].

**Table 1:** Blueprint of key components in the process evaluation.

Description of the components	Questions in the process evaluation	Data sources
**Context**		
*Contextual conditions for the implementation*	**Contextual barriers to and facilitators for reaching and retaining the target group**: What were the contextual barriers to and facilitators for reaching and retaining the target group?	Focus group discussions [FGDs] with group leaders and internal facilitators
	**Contextual barriers to participation of the group leaders**: What were the contextual barriers to participation for the group leaders?	Field notes
**Implementation process**		
*How delivery of the implementation is achieved*	**Reach**—**the extent to which the target group was reached and participated in the intervention**: To what extent did parents in the target group participate in the intervention? **Adaptation of the implementation strategy**: What adaptions of the implementation strategy were made, and how was the strategy perceived by group leaders and internal facilitators? **Fidelity**—**the extent to which the Ladnaan programme was delivered as intended**: To what extent was the Ladnaan programme delivered as intended, and how did group leaders perceive the delivery?	Attendance list Field notes Field notes FGDs with group leaders FGDs with internal facilitators Participant observations during societal information Reflection notes from lecturer FGDs with group leaders FGDs with Connect instructors Reflection notes from lecturer FGDs with group leaders
**Mechanism of impact**		
*How participants respond to the implementation strategy*	**Group leaders’ satisfaction with the delivery**: Were group leaders satisfied with the delivery of the intervention?	FGDs with group leaders
	**Parent satisfaction with the programme**: Were the participants/parents satisfied with the intervention?	Client satisfaction instrument to parents

This study has received ethical approval from the Regional Ethical Review Board in Uppsala, Sweden (Dnr 2014/048/1). Participants have received an information letter before participating and gave written consent (in-person interview) and verbal consent (phone interview) to participate. At any time during data collection, participants were allowed to decline from answering any questions or withdraw from the study without consequence.

### Setting

The study was conducted in a medium-sized municipality in Sweden with a population of 51,000. By the end of 2017, 17% of the municipal population was foreign born [36], with Somalis making up one of the largest immigrant groups. The municipality’s social services experienced considerable difficulty reaching out to immigrant parents and were determined to find ways to engage hard-to-reach groups in parenting programmes [37]. Therefore, this study was conducted in collaboration with social services.

### Intervention

**Fig 1:**
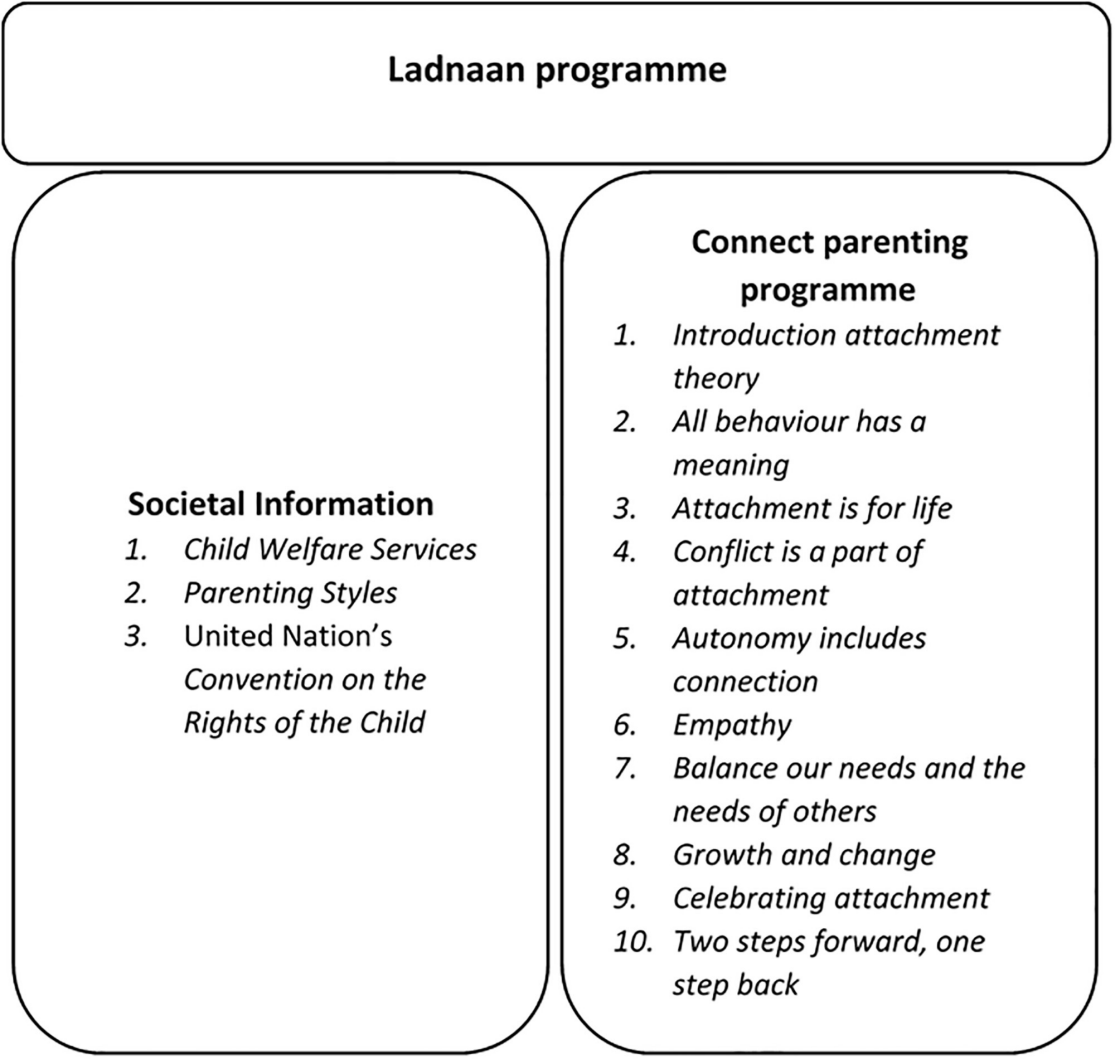
Components of the Ladnaan intervention.

As mentioned above, the Ladnaan intervention has two components: the Connect parenting programme [26] and societal information (Fig 1). The Ladnaan intervention was delivered four times to four separate groups, and each programme had 12 sessions, which were attended by 12–17 participants and delivered by two group leaders (one female and one male).

Connect is a 10-session parenting programme derived from attachment theory [38]. Its primary aim is to promote children’s mental health and strengthen the parent–child attachment relationship [38]. The programme was chosen based on findings from previous research [15] showing that Somali-born parents expressed a need to strengthen their relationships with their children. Each Connect session lasted for one hour, and each societal session lasted two to three hours. The Connect programme comprises 10 sessions based on the nine following attachment principles: 1) all behaviour has a meaning; 2) attachment is for life; 3) conflict is a part of attachment; 4) autonomy includes connection; 5) empathy is the heartbeat of attachment; 6) our needs and those of others must be balanced; 7) growth and change are part of a relationship; 8) celebrating attachment; and 9) two steps forward, one step back (see Fig 1). In each session, parents are introduced to one attachment skill. Group leaders use reflections, case examples, role plays and exercises to illustrate the attachment principles in-depth. After each Connect session, parents receive translated handouts summarising the session. For the Ladnaan intervention, the core component of the Connect parenting programme was not modified. However, the examples and some role plays were altered to make them more relatable to the target population. Somali metaphors and proverbs were also used during the sessions to make the content comprehensible and to clarify certain content.

The societal information component comprised two of the 12 sessions of the Ladnaan programme and was developed from a previous study on the need among Somali immigrant parents for parenting support [15]. The societal information component of the current programme contained the following three parts: 1) child welfare services, which provided parents with an overview of Swedish Child Welfare Services; 2) parenting styles, which introduced different parenting styles, such as democratic and authoritative parenting; and 3) the United Nation’s Convention on the Rights of the Child (CRC), which gave parents knowledge about the international human rights of children and the promotion of children’s agency. The societal information sessions were delivered as workshops in which group leaders briefly introduced the theme and proceeded with discussions and questions from the parents.

All group sessions for this study were conducted in Somali except for the single session on parenting styles, which was delivered by a Swedish-speaking professional from Family and Child Welfare Services and instantaneously translated by one of the group leaders. The Ladnaan intervention was delivered using a culturally sensitive approach by group leaders who had cultural competence related to both Swedish and Somali society. In this study, cultural sensitivity refers to the extent to which the target group’s language and experiences are incorporated into the design, delivery and evaluation of the intervention [39]. To engage parents in the intervention, the programme was named using the Somali word ‘Ladnaan’, and the term ‘parent empowerment’ was used to indicate that the programme was intended to empower parents rather than control them.

### Implementation strategies

The implementation strategies focused on the implementation of the Ladnaan programme, the recruitment and training of group leaders and lecturers and the reaching and retaining of parents, all of which were guided by the study’s culturally sensitive approach.

#### Recruitment and training of group leaders and lecturers

The main task for group leaders was to deliver the Connect sessions. Initially, nine group leaders (five males and four females) from Somali backgrounds employed by social services, the Integration Office for Newly Settled Refugees and schools were recruited to ensure that the parenting programme’s sustainability even after the study’s conclusion (Table 2). Each session had group leaders of a mix of genders (one female and one male) to attract both mothers and fathers to the programme—an initiative based on a prior study and meetings with Somali associations in the municipality. The group leaders received four days of standardised Connect training provided by Connect instructors qualified to train group leaders [38]. Two group leaders and one lecturer from the municipal Family and Child Welfare Services department delivered the societal information sessions.

**Table 2:** The study participants’ characteristics, areas of responsibility and workplace.

	Area of responsibility in the intervention	Workplace	Educational background
Group leaders* **[*n* = 8]**	Delivering the Connect programme Help in reading and writing letters from government agencies	**Social Services**: Department of Family, Children and Youth	University degree [*n* = 1]
		**Social Services**: Department of Employment and Integration	Upper secondary diploma [*n* = 1]
		**Integration office**	University degree [*n* = 1]
			Upper secondary diploma [*n* = 1]
		**Schools**: primary and secondary school	University degree [*n* = 1]
			Upper secondary diploma [*n* = 2]
		**Interpreter**	Upper secondary diploma [*n* = 1]
Lecturer* **[*n* = 1]**	Delivering the societal information component	**Social Services**: Department of Family, Children and Youth	University degree [*n* = 1]
**Connect instructors [*n* = 2]**	Training the group leaders in the Connect programme Supervising group leaders once a week in the Connect programme	**Social Services from another municipality—external quality assurance**	University degree [*n* = 2]
**Internal facilitators [*n* = 2]**	Sending text messages and calling parents Babysitting Taking care of the welcoming refreshments for the participants Arranging transportation	**Social services**	Vocational training diploma [*n* = 2]
**External facilitator [*n* = 1]**	Overall responsibility for the implementation process Observing the societal information sessions Quality assurance of the intervention	**University**	University degree [*n* = 1]

*Two of the group leaders also served as lecturers and delivered the societal information component. One group leader was also responsible for recruiting parents and facilitating the implementation of the intervention.

#### Strategy for reaching and retaining the target group

Strategies for reaching and retaining parents included the following components: 1) informal information meetings (held in small groups and at the individual level); 2) a diploma for parents who participated in the parenting programme; 3) times for meetings adapted to the parents’ needs; 4) a venue in the neighbourhood where most of the families lived; 5) reminders; 6) babysitting services; and 7) assistance with reading and answering letters from government agencies.

Parents first received information about the parenting programme at informal meetings held in Somali associations and schools and at the Workers’ Educational Association. The meetings were arranged together with the Somali associations and key individuals (Somalis from the municipality who were well respected within the Somali community). Parents interested in receiving more information were phoned by the external facilitator (member of the research group), who gave them detailed information about the parenting programme.

The key individuals and the external facilitator suggested that awarding a diploma to parents who completed the parenting programme could be a way to facilitate recruitment; therefore, parents were informed of this incentive. The sessions were held in the late afternoon at a time and place chosen by the parents in a neighbourhood in which most of the parents lived.

Text-message reminders were sent to parents by internal facilitators the day before each session. Childcare was offered to parents who required it to increase the chances of both parents attending the sessions. Parents were also offered assistance with reading and replying to letters from authorities. According to the Somali associations, many parents in the community often sought assistance in reading government letters.

### Study participants

Those involved in the study comprised the group leaders, a lecturer, Connect instructors and the internal facilitators who aided in the implementation process (Table 2), as well as the parents who participated in the parenting programme. The participants involved in implementing the programme included eight females and six males, excluding the parents. Their roles and characteristics are described in Table 2.

The parents who participated in the parenting programme (*n* = 58) were between 30 and 70 years old and had children aged 11 to 16 years old. The majority (65%) of the parents had lived in Sweden for less than five years. One participant was lost to follow up, therefor 58 participants were included in this study.

### Data collection

For this process evaluation study, both qualitative and quantitative data were collected and analysed [40] (see Table 3 for data collection sources, participants and data analysis). The combination of qualitative and quantitative data allowed for a focus on different aspects of the programme’s implementation. Data were collected during the delivery of the four rounds of the programme and after its completion.

**Table 3:** Data collection sources, participants and data analysis.

Data source	Participants/instruments	Data collection	Data analysis
FGDs with Connect instructors, group leaders and internal facilitators [*n* = 6]	Group leaders who delivered the intervention [*n* = 7]	FGDs were conducted after the four parent groups were finished	Inductive qualitative analysis
	Internal facilitators responsible for practicalities [*n* = 2] concerning the implementation		
	Instructors of the Connect programme [*n* = 2]		
Field notes	Field notes of the external facilitator during planning, delivery and evaluation phases	Field notes were made during the delivery of the programme	Deductive qualitative analysis
Participant observation	Participant observation of the external facilitator during the societal information sessions [*n* = 2]	Observations were conducted during the delivery of the programme	Deductive qualitative analysis
Reflection	Reflection notes from the two group leaders and the lecturer	Reflections were made after the delivery of the societal information sessions	Deductive qualitative analysis
CSQ [*n* = 57]	CSQ is a standardised instrument measuring parents’ satisfaction with the programme	The questionnaire was administered two months after the programme ended	Descriptive statistical analysis
Attendance list	Attendance list collected from all the sessions [*n* = 12] in the programme	Attendance list was noted at all sessions	Descriptive statistical analysis

#### Qualitative methods of assessment

The qualitative data were collected through focus group discussions (FGDs), field and reflection notes and observations of the societal information sessions. A semi-structured interview guide was developed covering the following themes: the group leader’s experiences of delivering the intervention, contact and interaction with participants, barriers and facilitators for implementation, how the intervention was implemented and delivered, and if the group leaders implemented the intervention as intended (see Table 4, S1 Table in S1 File, the topic guide used in this study). The interview guide was tested with the first FGD. No further revisions to the guide were made. Six FGDs were conducted with group leaders, a lecturer, Connect instructors and internal facilitators. The FGDs lasted from 60 to 105 minutes. Five FGDs were conducted in Somali and one in Swedish.

**Table 4:** Topic guide for focus group discussions for group leaders, internal facilitators and Connect instructors.

Target group	Topic	Questions
*Group leaders*, *lecturer*, *internal facilitators*	***Training and supervision***	What was your experience of the Connect training?
		Did it provide you with enough knowledge and preparation to lead the intervention?
		What was your experience of the supervision meetings?
	***Delivery of the programme***	Can you tell me about your experience delivering the Ladnaan intervention?
		How secure were you in delivering the intervention?
		Follow-up questions regarding the different sessions. Were the sessions delivered as per the manual?
		If you think back to parent sessions, how did the parents receive the intervention as a whole? Were there any particular sessions that were more positive than others? Which sessions? Why?
		Delivery time: Were the different parts of the intervention carried out as planned? How was the time for delivering the sessions?
		How was the interaction between you and the parents?
		How was the interaction between the two group leaders?
	***Barriers and facilitators***	What obstacles did you encounter in delivering the parenting intervention? *Programme*, *organisation*, *support from employers*, *supervision*
		What factors have facilitated the delivery of the intervention?
		If you were to say that any particular successful factor was an important ingredient in the implementation, which would it be?
	***Relevance***	Does the societal information cover the needs of Somali immigrant parents? What more would they need?
		Does the Connect programme cover the needs of Somali immigrant parents to improve parent–child relationships? What more would they need?
*Connect instructors*	***Training and supervision***	What were your experiences in training the group leaders on Connect?
		Were they sufficiently equipped to deliver the intervention? How?
		What were your experiences with the supervisions?
	***Fidelity of the programme***	Has the intervention been delivered as in the manual?
		We encouraged group leaders to culturally adapt role plays and exercises; what was your experience?
		Were there any specific role plays or exercises that the parents did not understand and group leaders had difficulty bringing about?

To capture the fidelity of the Connect programme, each session was video-recorded, and the recordings were sent to the Connect instructors. For this study, we did not analyse the video recordings but held FGD with the instructors who supervised the group leaders each week throughout the sessions. The Connect instructors were asked about their experiences of delivering training and supervising the group leaders, as well as about the fidelity of the programme. The FGDs with the instructors were conducted in Swedish and lasted approximately 60 minutes.

Moreover, to capture the fidelity of the societal information sessions, two observations and reflection notes from three leaders of the societal information sessions were collected. For this purpose, observation and reflection schemes were developed. Both schemes contained similar information and were about overall impressions and interactions with the parents, the most common participant questions and session duration. Each lecturer who delivered two sessions wrote a reflection regarding the delivery and interactions with the parents (see Table 5, the observation and reflection scheme).

**Table 5:** Observation and reflection note scheme.

Topic	Questions
***Environment***	Describe the venue for the sessions—how did it look?
***Participants***	Who participated in the sessions? Gender, positions.
***Societal information sessions***	How well were the societal information sessions delivered?
	Were the key points related to the sessions delivered as intended?
***Interaction between group leaders and parents***	Did communication happen in a supportive and clear manner?
	Were the educational and supportive aspects balanced in the session?
	What questions and thoughts did participants commonly have?
	What was discussed the most? What questions sparked discussion?
***Time for delivery***	What was the session duration? Were the sessions delivered as intended? What was left out?

Field notes were taken during the implementation of the parenting programme to examine the contextual factors that facilitated or hindered the implementation. The focus for the field notes was to capture how the components in the implementation strategy worked as facilitators and barriers in the implementation process.

#### Quantitative methods of assessment

The quantitative data included a questionnaire and an attendance list. The Consumer Satisfaction Questionnaire (CSQ) was used to measure parents’ satisfaction with the intervention and was administered to participants two months after intervention completion [41]. The CSQ is a standardised instrument that was adapted for this study, meaning that the questions focused on the current intervention. The questionnaire was also translated into Somali in accordance with the following five steps of the translation process [42]: 1) forward translation, 2) backward translation, 3) expert panel back-translation, 4) pre-testing and 5) final version. The CSQ comprised 22 items addressing the impact of the parenting programme on the parents’ knowledge, confidence with parenting and parent–child interactions and relationships. Parents were asked to rate each item on a scale from 1 (e.g. satisfaction with the programme or improvement of problems or parent–child relationships) to 4 (dissatisfaction with the programme or worsened problems or parent–child relationships). The 22 items were summarised to yield a total score between 22 and 88, with low scores indicating higher satisfaction. Dose was assessed using the attendance list.

### Data analysis

All FGDs were transcribed verbatim and analysed inductively with content analysis [43]. This method was chosen to understand how the participants perceived and experienced the phenomenon (i.e. implementation strategies). The transcribed FGDs with the Connect instructors were analysed separately. All transcribed data were read by the first author several times to ascertain the participants’ overall experiences. Phrases, paragraphs and words that captured the key concepts were then highlighted and assigned initial codes. The next step involved sorting codes based on their similarities. The co-authors reviewed the coding, and when consensus was achieved, the codes were sorted according to the components of the process evaluation guidance.

The field notes, observations and reflection notes were analysed using deductive content analysis [43]. The authors started by reading the text and then placed the text according to the components of the process evaluation guidance.

The CSQ was analysed in SPSS version 24. Descriptive statistics (frequencies and percentages) were calculated to describe the data.

## Results

The results of the process evaluation were outlined using the framework developed by Moore et al. [32], taking into consideration the main components of contextual condition, implementation and mechanism of outcome (see Table 1). Quotes were used to illustrate the qualitative FGDs, and participants’ names were kept confidential through the use of the abbreviation FR (female respondent) or MR (male respondent).

### Contextual conditions for the implementation

#### Contextual barriers to and facilitators for reaching and retaining the target group

According to the interviews with the group leaders and the field notes, one contributing factor to the implementation was the involvement of key individuals who were well-known Somalis and respected within the community. These key individuals arranged and participated in initial informal meetings and reassured parents of the benefits of participating in the programme. The group leaders and internal facilitators also stated that the mixed gender of the group leaders contributed to fathers being reached and retained for the sessions. The group leaders and external facilitators emphasised that their own positions were crucial in terms of the cultural competence that the programme required. One group leader stated, ‘It was having the right person behind each post/job that we were very grateful for’ (FGD 4 FR 2).

The internal facilitators noted that parents were hesitant about the involvement of social services in their parenting and in the delivery of the intervention, making the involvement of social services a potential barrier to reaching and retaining parents in the programme. Therefore, it was important to start with the societal information sessions, as one of the lecturers who later led the Connect programme stated:

*It was crucial that we began by talking about social services work with children and youth*. *This gave parents an understanding of the true purpose of social services and also helped relieve them of some of their worries*. *It also facilitated the engagement of parents in other topics in the parenting programme*. *I think we should always start with what is important to parents or what concerns them*.

(FGD 1, FR1)

The internal facilitators stressed that the venue was unsuitable for more than three children and lacked toys, which may have prevented couples from bringing their children and thereby from participating in the programme together. The facilitators suggested finding a more suitable and child-friendly venue where both young and older children could play during the sessions.

#### Contextual barriers to the group leaders’ participation

An additional contextual factor that threatened the group leaders’ ability to deliver the intervention was the lack of managerial support. Some group leaders reported that they lacked support from their manager, while group leaders who worked at schools reported that they did not have enough preparation time for the sessions. The group leaders suggested that the managers should be involved in the planning phase so that they could set aside time for the group leaders to prepare and deliver the sessions. The group leaders estimated needing 8–10 hours per week for preparation and delivery.

The group leaders emphasised that programmes become sustainable when funded by the municipality or civil society. They believed that if the Somali associations (and not social services) had implemented the parenting programme, it might have been easier to recruit parents but more difficult in terms of sustainability and quality assurance.

### Implementation process

#### Reach—The extent to which the target group was reached and participated in the intervention

A total of 60 parents were invited to participate in the programme, and 58 participants completed the programme. Two parents could not attend any sessions due to sickness or work. Of the 58 participating parents, nearly one-third were fathers (*n* = 17). Only two families arrived with both fathers and mothers. Forty participants attended more than eight sessions, while 17 attended fewer than eight sessions. The mean attendance for all 12 sessions was 8.01 (SD = 3.4) sessions. A majority of the participants (*n* = 40, 70%) took part in the two societal information sessions, seven participants (12%) took part in only one session and eleven participants (18%) did not attend any of the sessions on societal information. For the Connect sessions, most of the participants (*n* = 40, 67%) took part in all 10 sessions.

#### Adaptations of the implementation strategy

The field notes showed that peer information (i.e. parents who completed the programme informing other presumptive participants about their experience) was added to the strategies to reach and retain parents after the first two groups completed their sessions. This strategy was added due to the difficulties of recruiting further parents to the programme. The two internal facilitators emphasised that peer information had contributed to reaching more parents.

According to the group leaders and facilitators, parents appreciated receiving a diploma upon successful completion of the programme, which was a strategy to retain their involvement.

Another implementation strategy aimed at retention was sending reminders to participants. However, it soon became clear that this strategy needed reinforcement, and, subsequently, internal facilitators offered parents living outside the neighbourhood free transportation, which contributed to high attendance. One internal facilitator said:

*In the beginning*, *when we saw that they [parents] hadn’t arrived 30 minutes before the session*, *we phoned them*. *If they told us that they had difficulties coming due to a lack of transportation*, *we offered them a lift*. *However*, *we later came to give all parents who lived outside the neighbourhood a lift*.

(FGD3, MR)

Many participants took advantage of the willingness of group leaders and facilitators to read and write their letters at most of the sessions. One group leader described his experience of supporting participants:

*It was positive supporting parents by reading or writing letters for them because the parents were stressed about all the letters they received*. *In addition*, *this kind of support was helpful in engaging and retaining the participants in the sessions*.

(FGD3, MR)

#### Fidelity—The extent to which the Ladnaan programme was delivered as intended

The interviews with the group leaders demonstrated that their training with other group leaders before and during the programme, along with the supervision, enhanced their competence and self-confidence in delivering the parenting programme.

Connect instructors reported that the group leaders were knowledgeable both during the Connect training course and delivery of the sessions. According to the Connect instructors, the group leaders delivered the programme according to the manual. The Connect instructors felt that the group leaders delivered the sessions with sensitivity, listened to the parents and sought to explain information in various ways. The Connect instructors also encouraged the group leaders to use metaphors and proverbs as well as verses from religion, as one Connect instructor explained:

*I encouraged them to use metaphors*, *proverbs and religious verses because we noticed that parents became engaged in the topic and had a lot to discuss when the group leaders used a proverb*, *something from the Quran or sometimes their own experiences*.

The Connect instructors pointed out that the supervision sessions were very important to ensuring that the programme was delivered as intended. The lectures on parenting style and on the work of social services were held in one session and took longer than planned. As a result of the FGD with the group leaders and the lecturer’s reflection notes, a suggestion was made to divide the two topics (societal information and parenting styles) into separate sessions and deliver the parenting styles lecture in Somali.

### Mechanism of impact

#### Group leaders’ satisfaction with the delivery

The group leaders reported that it took two to three sessions to build trust with parents and for parents to accept the programme. According to the group leaders, at the beginning, the parents were hesitant and concerned about the motives behind the involvement of social services; some parents denied that they needed training in their parenting. However, some group leaders stated that most of the parents and group leaders knew each other, which facilitated the building of trust with the parents. Another crucial factor that contributed to the retention of participants was the societal information component of the Ladnaan programme because most parents were eager to receive this information.

The group leaders agreed that having Somali as their mother tongue and that of the parents increased parental involvement, which, in turn, contributed to the parents completing the programme. The group leaders also felt that their shared cultural background with the parents was a contributing factor to success. As one group leader stated:

*I would say that the biggest success factor was that the group leaders and the parents shared the same country*, *culture and language and understood each other… for instance*, *a Swedish group leader would present the message*, *and I would add examples from the culture or examples that they could identify with*.

(FGD3, FR)

The use of poetry, proverbs and metaphors served to make the programme culturally sensitive, which seemed to help the parents understand, recognise and realise the universality of parenting. The group leaders noted that the adaptation of role-play exercises and examples was crucial for parents to understand and view the parental–child interaction from different perspectives, leading to further reflections and discussions by the parents.

#### Parent satisfaction with the programme

Parents were asked about their satisfaction with the Ladnaan programme, and the CSQ showed that 96% (*n* = 55) of the participants were very satisfied (see Table 6). Most (70%) reported increased knowledge about social services and children’s rights and increased confidence in seeking support from social services when facing difficulties with their children. All participants (*n* = 57) stated that their relationships with their children had improved after completing the course. They reported a better post-programme understanding of themselves as parents and of their children’s needs and behaviour and indicated feeling more secure in their parenting role in Sweden. According to the CSQ (*n* = 39), the total satisfaction ranged from 24 to 43 (*M* = 26.38, *SD* = 4.13), which indicates that families were highly satisfied with the programme.

**Table 6:** Parent satisfaction with the programme according to the Client Satisfaction Questionnaire (CSQ) (*n* = 57).

Variables	*n* (%)
**Total satisfaction M**^1^ **(SD**^2^**, min–max)**	26.38 (4.13, 24–43)
Missing	18 (32)
**Satisfied with the overall programme**	
Very satisfied	55 (96)
Somewhat satisfied	2 (4)
Not satisfied	0
Missing	0
**Satisfaction with the societal information sessions**	
Very satisfied	42 (73.6)
Somewhat satisfied	4 (7.1)
Not satisfied	1 (1.8)
Missing	10 (17.5)
**Increased knowledge about social services**	
Very much	40 (70.2)
Somewhat	6 (10.5)
Not really	1 (1.8)
Missing	10 (17.5)
**Confidence in seeking support from social services**	
Very much	42 (73.6)
Somewhat	4 (7.1)
Not really	1 (1.8)
Missing	10 (17.5)
**Satisfaction with the Connect programme sessions**	
Very satisfied	49 (85.9)
Somewhat satisfied	8 (14.1)
Not satisfied	0
Missing	0
**Increased parent reflection on child’s behaviour due to Connect programme**	
Very much	52 (91.1)
Somewhat	4 (7.1)
Not really	1 (1.8)
Missing	0
**Feeling more secure as a parent due to Connect programme**	
Very much	50 (87.7)
Somewhat	7 (12.3)
Not really	0
Missing	0
**Current parent–child relationship compared to 6 months ago**	
Much better	50 (87.7)
Little better	4 (7.1)
Same	3 (5.2)
Missing	

^1^ M = mean,

^2^ S = standard deviation

## Discussion

Overall, our results showed that the most important contextual factor that facilitated reaching and retaining Somali-born parents in Sweden was the involvement of key individuals. These individuals built trust with the Somali community, which contributed to reaching and retaining parents in the programme. In addition to the implementation strategies used prior to delivery, further implementation strategies were used to reach and retain participants during the programme. For instance, peer information was employed to recruit more parents. To retain participants, parents were offered free transportation. The overall findings also showed that the programme was implemented and delivered as intended. A majority of the parents were satisfied with the programme and reported increased knowledge of children’s rights and the support they could seek from social services. They also acknowledged an improvement in the parent–child relationship.

Given previous reports of involving key individuals who are trusted and known by the target group [24], our study showed that the success of the programme was the contextual adaptability, cultural sensitivity and a trusting relationship. The trusting relationship was facilitated by the involvement of key individuals within the Somali community in the early phase of the study, which enabled reaching, recruiting and retaining Somali-born parents in the programme. Moore et al. [32] emphasise the importance of understanding and taking into consideration the contextual factors that may impact the implementation and outcome of the programme. Crucial to the parents engaging in the programme was their understanding of the benefits to them and their children and the face-to-face provision of information at meetings. During participant recruitment, several challenges regarding reach of parents arose, so adding peer information facilitated by parents who had already participated in the intervention contributed to reaching more parents. The programme was delivered by two group leaders, one female and one male, and this strategy contributed to reaching and retaining more fathers. This study included more fathers compared to other studies [44]. Therefore, to attract both mothers and fathers, it may be beneficial to have group leaders of both genders deliver the programme. Parenting in Sweden jeopardised traditional gender roles for both Somali-born fathers and mothers, with fathers feeling a loss of authority in the family and mothers experiencing inadequacy as mothers due to societal expectations that they also work or study outside the home [15]. Having access to both a male and a female leader facilitated a discussion on these issues and a reconstruction of gender expectations and parenting in the new country.

A contextual factor for the specific target group was that Somali-born parents who had arrived recently in Sweden were unfamiliar with the function of authorities and afraid of the legal authority that, for example, social services had to apprehend their children [7, 14, 15]. Parents in this study reported that their increased knowledge of the social services’ work contributed to their confidence in seeking support from them. It may be hypothesised that the societal information component supported parents’ satisfaction with and high attendance at the programme because it addressed specific needs Somali-born parents had. This is in line with our previous study in which we interviewed Somali-born parents on their experiences of participating in the Ladnaan programme [28]. Offering parents information about their responsibilities and rights as parents in the new country, as well as about the rights of their children, can contribute to empowering them in their role as parents [28].

Our findings indicate the benefits of having social services deliver the parenting programme, which led to parents having less fear and increased confidence when seeking support from social services. Social services’ involvement in the intervention can also contribute to quality assurance and the sustainability of support services for the most vulnerable parents. A contextual factor that needs to be considered in terms of sustainability and quality assurance is involving group leaders within the municipality because this will ensure sustainability and trust-building with the community. Another contextual factor is ensuring that group leaders receive time for preparation. In this study, some group leaders prepared for the sessions in their spare time.

As we know from the literature, the adaptation of interventions to different contexts may ensure greater acceptance of the programme among participants [24, 45]. However, the adaptation must consider contextual as well as cultural aspects while maintaining the core components of the interventions. In this study, the programme was designed and delivered in a culturally sensitive manner in the participating parents’ native language by Somali group leaders who had similar backgrounds to the parents. The group leaders in this study highlighted the importance of delivering parenting programmes in the parents’ native language and having group leaders with contextual and cultural competence. Several studies underscore the importance of cultural sensitivity in the interventions [23, 24, 46, 47], highlighting that parenting programmes given in the participants’ native language and tailored to their needs are particularly effective [23, 24]. In this study, the group leaders drew upon their own experiences and used Somali metaphors and proverbs to emphasise certain elements of the programme, facilitating not only understanding but also a sense of familiarity and equality.

The retention of parents in the programme was improved as a result of the cultural awareness of the facilitators and group leaders. Our findings showed that most parents attended eight sessions or more—a retention level that might be linked to the parents’ satisfaction with the programme. As mentioned above, the programme was designed according to the parents’ needs and delivered in their native language by people who shared the same cultural background as parents. Moreover, any hindrances or stressful factors that could result in parents not attending the sessions were eliminated by offering them childcare and convenient session times. Previous studies [23, 24, 48] have reported on the practical issues that may prevent parents from participating in parenting programmes, such as transportation, childcare, inconvenient meeting times and other stressful factors. The parents in our study were offered childcare services during the sessions, but few used them. The study demonstrated the importance of offering childcare in a child-friendly venue with age-appropriate activities for the children. The phone reminders, assistance with reading government correspondence and transport to the sessions were reported to reduce parental stress.

### Limitations of the study

The data collection was carried out by the first author (FO), who was also involved in developing the programme. However, the analysis and interpretation of the study were carried out by the entire research group, who continuously discussed the data analysis and interpretation of results. A limitation concerning parents is that they were only interviewed through the satisfaction questionnaire; additional data on their perceptions of the programme and its usefulness would have illuminated the process further. However, the researchers have previously reported parents’ experiences on the parenting intervention [28].

The programme fidelity was assessed through interviews with the instructors, who evaluated the recorded sessions. It would have been preferable to assess the recorded sessions using a checklist. However, during the delivery of the intervention, the external facilitator, researchers and instructors held weekly meetings to ensure that the programme was delivered according to the manual. While the contextual factors of this implementation process relate to only one municipality, they are in line with previous international research [23, 24, 48]. Nevertheless, this is a limitation of this study, and studies should be conducted on the delivery of this type of programme in major cities and with other groups.

## Conclusion

This study provides a clearly described strategy for the implementation of a parenting programme and guidance on how to reach and retain immigrant parents for such programmes. We found that contextual factors, such as matching the group leaders and the parents (shared language) and cultural awareness, are important when it comes to building trust with immigrant parents and engaging them in parenting programmes. Another crucial factor with wider applicability to other immigrants is designing and delivering parenting programmes in a culturally sensitive manner. When implementing evidence-based practice in the delivery of social services, it is important to consider both the specific group for which the intervention is intended and any prevailing contextual factors. The fact that social services delivered the parenting programme gave Somali-born parents the confidence to seek support from social services. The findings may inform policies by providing an example of how to adapt and deliver evidence-based programmes to immigrant groups coming to high-income countries. The experiences from the process evaluation also provide transferrable knowledge on how to reach hard-to-reach groups.

## Supporting information

S1 File Topic guide in English and Swedish. (DOCX)

## Abbreviations

CRC: Convention on the Rights of the Child
CSQ: Consumer Satisfaction Questionnaire
FGD: Focus group discussion
FR: Female respondent
MR: Male respondent
