# Supplementary material for: The implementation of a culturally tailored parenting support programme for Somali immigrant parents living in Sweden—A process evaluation
Source: PLoS One. 2022 Sep 14;17(9):e0274430. doi: 10.1371/journal.pone.0274430 (PMC9473391; doi:10.1371/journal.pone.0274430)
Supplement: S1 File — (DOCX) [file pone.0274430.s001.docx]

**The implementation of a culturally tailored parenting support programme for Somali immigrant parents living in Sweden – a process evaluation**

Fatumo Osman^1*^, Ulla-Karin Schön^1, 2^, Marie Klingberg-Allvin^1^, Renée Flacking^1^, Malin Tistad^1^

^1^ Department of Health and Welfare, Dalarna University, Falun, Sweden

^2^ Department of Social Work, Stockholm University, Stockholm, Sweden

**S1 Table. Topic guide in English: focus group discussions for group leaders, internal facilitators, connect instructors**

| **Target group** | **Topic** | **Questions** |
| --- | --- | --- |
| *Group leaders, lecturer, internal facilitators* | **Training and supervision** | What were your experiences of the Connect training?  Did it provide you with enough knowledge and preparation to lead the intervention?  What was your experiences of the supervision meetings? |
|  | **Delivery of the programme** | Can you tell me about your experience delivering the Ladnaan intervention?  How secure were you in delivering the intervention?  Follow-up questions regarding the different sessions.  If you think back to parent sessions, how did the parents receive the intervention as a whole? Were there any particular sessions that were more positive than others? Which sessions? Why?  Delivery time: Were the different parts of the intervention carried out as planned? How was the time for delivering the sessions?  How was the interaction between you and the parents?  How was the interaction between the two group leaders? |
|  | **Barriers and facilitators** | What obstacles have you encountered in delivering the parenting intervention? *Program, organization, support from employers, supervision*  What has facilitated delivering the intervention?  If you were to say any successful factor has been an important ingredient in the implementation, what was it? |
|  | **Relevance** | Does the societal information cover the needs of Somali immigrant parents? What more would they need?  Does the Connect programme cover the needs of Somali immigrant parents on improving parent-child relationships? What more would they need? |
| **Connect instructors** | **Training and supervision** | What were your experiences in training the group leaders on Connect?  Were they sufficiently equipped to deliver the intervention? How?  What were your experiences with the supervisions? |
|  | **Fidelity of the programme** | Has the intervention been delivered as in the manual?  We encouraged group leaders to adapt role-plays and exercise culturally, what was your experiences?  Were there any specific role-plays or exercises that the parents did not understand and group leaders had difficulty bringing about? |

**S2 Table. Topic guide in Swedish: focus group discussions for group leaders, internal facilitators, connect instructors**

| **Målgruppen** | **Tema** | **Frågor** |
| --- | --- | --- |
| *Gruppledare, föreläsarna samt facilitatorer* | **Utbildning och handledning** | Vilka var dina erfarenheter av Connect utbildningen?  Hur säker var du på att bedriva programmet efter utbildningen? Kände du dig att du var utrustat med kunskap som behövdes för att bedriva den?  Vilka är dina erfarenheter av hur handledningen har fungerat? |
|  | **Leverera programmet** | Kan du berätta om dina erfarenheter av att ha lett föräldrakursen Ladnaan?  Hur säker var du på att bedriva programmet? Uppföljningsfrågor kring de olika sessionerna.  Om du tänker tillbaka på föräldraträffarna, hur har föräldrarna tagit emot kursen som helhet? Fanns det vissa principer/ämne som har varit mer positiva än andra för föräldrarna? Vilka? Varför?  Tid för att leverera: Hur såg tiden ut för sessionerna? Har tiden räckte till eller skulle du ha behövt mer tid? Kunde du leverera enligt manualen?  Hur var interaktionen/samspelet mellan dig och föräldrarna?  Kan du berätta om samspelet mellan dig och din kollega som du har ledd kursen tillsammans? |
|  | **Barriärer och möjligheter** | Vilka hinder har du stött på för att bedriva föräldrakursen? *Program, organisation, stöd från arbetsgivare, handledning.*  Vad har underlättat för att bedriva föräldrakursen?  Om du skulle säga någon framgångsrikfaktor som har varit viktigt ingrediens i intervationen, vad var det? |
|  | **Relevansen** | Täcker tillägget föräldrautbildningen somalisk-födda föräldrars behov? Vad mer skulle de ha behövt?  Täcker Connect programmet somalisk-födda föräldrars behov? Vad mer skulle de ha behövt för att förbättra föräldra-barn relationen? |
| **Connect handledarna** | **Utbildning och handledning** | Vilka är dina erfarenheter med de fyra dagars utbildning till gruppledarna?  Var gruppledarna tillräckligt utrustade för att leverera interventionen efter utbildningen? På vilket sätt?  Vilka är dina erfarenheter av handledningen? |
|  | **Programtrogenhet** | Har gruppledarna levererat programmet enligt manualen?  Vi uppmuntrade gruppledarna att kulturanpassa rolespelen och övningar, har de kulturanpassad rollspelen eller annat i manualen? Vad? Hur blev det?  Fanns det några specifika rolespel eller övningar som föräldrarna inte förstod och gruppledarna hade svårt att förklara det? |
